# Clozapine-Induced Pericardial Effusion: A Case Report

**DOI:** 10.1192/j.eurpsy.2026.12161

**Published:** 2026-07-31

**Authors:** G. G. Cetin, E. N. Keskinege, S. Kiraz, E. Göka

**Affiliations:** Psychiatry, Ankara Bilkent City Hospital, Ankara, Türkiye

## Abstract

**Introduction:**

Clozapine is an atypical antipsychotic with a unique side effect profile, including serious adverse events such as agranulocytosis, seizures, myocarditis, and pericarditis. Although rare, pericardial effusion has also been reported and may pose a life-threatening risk.

**Objectives:**

To highlight pericardial effusion as a rare but significant adverse effect of clozapine and to monitor its clinical course.

**Methods:**

A case of clozapine-induced pericardial effusion in a patient with treatment-resistant schizophrenia is presented.

**Results:**

The patient was admitted to the psychiatry ward with depressive symptoms, impaired self-care, social withdrawal, and grandiose and persecutory delusions. She had a thirty-year history of treatment-resistant schizophrenia and had been hospitalized five times, having received paliperidone, olanzapine, aripiprazole, and clozapine. At admission, she was not receiving psychiatric treatment and had no history of systemic illness.

During hospitalization, paliperidone and haloperidol were initiated but were ineffective and caused extrapyramidal symptoms. The patient was re-evaluated and clozapine was gradually titrated. At a dose of 100 mg/day, she developed tachycardia (110-120 beats per minute) and orthostatic hypotension, though remained afebrile. Laboratory tests, including complete blood count, biochemical markers, cardiac enzymes, and electrocardiography, were normal except for tachycardia, and no infectious focus was identified.

Before clozapine initiation, she had undergone abdominal ultrasonography for nausea, vomiting, and right upper quadrant pain. Magnetic resonance cholangiopancreatography revealed incidental bilateral pleural effusion. Pulmonology consultation was obtained, and computed tomography pulmonary angiography demonstrated pericardial effusion. Transthoracic echocardiography confirmed a 2 mm pericardial effusion adjacent to the right ventricle, without hemodynamic compromise.

Clozapine was titrated to 200 mg/day per treatment guidelines. In the absence of prior cardiac pathology, a drug-induced cause was suspected. Cardiology advised daily monitoring with electrocardiography, troponin, acute phase reactants and follow-up transthoracic echocardiography. Despite clinical stability, the effusion persisted and gradual discontinuation of clozapine was considered.

**Conclusions:**

Pericardial effusion is a rare but potentially serious complication of clozapine. Early detection and multidisciplinary care are vital. This case underlines the importance of cardiac monitoring during clozapine treatment, particularly when unexplained cardiac symptoms occur.

**Disclosure of Interest:**

None Declared

